# Identification of the Stapled α-Helical Peptide ATSP-7041 as a Substrate and Strong Inhibitor of OATP1B1 In Vitro

**DOI:** 10.3390/biom13061002

**Published:** 2023-06-16

**Authors:** Rika Ishikawa, Kosuke Saito, Takashi Misawa, Yosuke Demizu, Yoshiro Saito

**Affiliations:** 1 Division of Medical Safety Science, National Institute of Health Sciences, Kawasaki 210-9501, Japan; ri-ishikawa@nihs.go.jp (R.I.); yoshiro@nihs.go.jp (Y.S.); 2 Division of Organic Chemistry, National Institute of Health Sciences, Kawasaki 210-9501, Japan; misawa@nihs.go.jp (T.M.); demizu@nihs.go.jp (Y.D.)

**Keywords:** ATSP-7041, MDM2/MDMX inhibitor, α-helical peptide, drug–drug interaction, organic anion transporting polypeptide 1B1

## Abstract

ATSP-7041, a stapled α-helical peptide that inhibits murine double minute-2 (MDM2) and MDMX activities, is a promising modality targeting protein–protein interactions. As peptides of molecular weights over 1000 Da are not usually evaluated, data on the drug–drug interaction (DDI) potential of stapled α-helical peptides remain scarce. Here, we evaluate the interaction of ATSP-7041 with hepatic cytochrome P450s (CYPs; CYP1A2, CYP2C9, CYP2C19, CYP3A4, and CYP2D6) and transporters (organic anion transporting polypeptides (OATPs; OATP1B1 and OATP1B3), P-glycoprotein (P-gp), and breast cancer resistance protein (BCRP)). ATSP-7041 demonstrated negligible metabolism in human liver S9 fraction and a limited inhibition of CYP activities in yeast microsomes or S9 fractions. On the contrary, a substantial uptake by OATPs in HEK 293 cells, a strong inhibition of OATP activities in the cells, and an inhibition of P-gp and BCRP activities in reversed membrane vesicles were observed for ATSP-7041. A recent report describes that ALRN-6924, an ATSP-7041 analog, inhibited OATP activities in vivo; therefore, we focused on the interaction between ATSP-7041 and OATP1B1 to demonstrate that ATSP-7041, as a higher molecular weight stapled peptide, is a substrate and strong inhibitor of OATP1B1 activity. Our findings demonstrated the possibility of transporter-mediated DDI potential by high molecular weight stapled peptides and the necessity of their evaluation for drug development.

## 1. Introduction

Peptides are an established modality for drugs; however, their usage has limitations, including the lack of conformational stability of their secondary structures, such as the α-helix, poor cell membrane permeability, and proteolytic instability. Therefore, efforts have been made to overcome these limitations by developing various types of macrocyclic peptides to expand their potential as drug candidates [1,2]. One of these types that have been extensively studied is stapled α-helical peptides, which represent a potential therapeutic modality targeting intercellular protein–protein interactions [3,4,5,6,7,8,9]. These peptides have a stable α-helical conformation and can bind to intercellular target proteins with high affinity and specificity [2,8,9], including murine double minute 2 (MDM2)/MDMX [10,11], BCL-2 family proteins [12], estrogen receptors [13], and β-catenin [14]. Among these, MDM2/MDMX, which negatively regulates p53 function, leading to tumor growth and proliferation, has been extensively studied. The stapled α-helical peptide ATSP-7041, the first potent dual inhibitor of MDM2 and MDMX activities, achieved effective suppression of tumor growth through the activation of the p53 pathway in p53 wild-type cancer cell lines derived from osteosarcoma and breast cancer in vitro and in vivo [15]. Furthermore, ATSP-7041 showed efficient cellular membrane penetration, extended half-life in blood/tissues, broad tissue distribution, and major elimination through bile [15]. The anti-tumor efficacy of ATSP-7041 has been validated in various cancer cell lines with wild-type p53 in vitro [16,17] and in vivo [18,19]. In addition, one of the analogs of ATSP-7041, ALRN-6924, has been evaluated in a clinical study for cancer therapy with a C_max_ value of 1.6–50 μM [20]. These findings suggest ATSP-7041 as a promising stapled peptide with a high potential to proceed to the clinical stage of drug development.

In drug development, the drug–drug interaction (DDI) potential of drugs should be evaluated as interactions with co-administered drugs alter the pharmacokinetic profile of the drugs and can cause serious adverse effects or reduce their therapeutic efficacy [21,22,23]. DDI occurs when a perpetrator drug induces or inhibits the activity of drug-metabolizing enzymes and/or transporters for a co-administered victim drug [24]. Therefore, in the evaluation of DDI potential, it is important to characterize the interactions of drugs with drug-metabolizing enzymes and transporters, including whether they are substrates and/or inhibitors. The well-characterized drug-metabolizing enzymes involved in DDI are cytochrome P450 (CYP) 3A4, CYP2Cs (CYP2C8, CYP2C9, and CYP2C19), CYP2D6, CYP1A2, and CYP2B6 [25]. Conversely, the well-characterized hepatic transporters involved in DDI are P-glycoprotein (P-gp), breast cancer resistance protein (BCRP), organic anion transporting polypeptide 1B1 (OATP1B1), and OATP1B3 [25]. DDIs via OATPs have a substantial effect, where the area under the curve of plasma concentration is potentially altered over five-fold [25]. To date, DDI potential evaluations have focused on low molecular weight (Mw) drugs, owing to the small Mw and size of the reported substrates and inhibitors as well as the substrate-binding pockets of drug-metabolizing enzymes and transporters [26,27,28]. Therefore, data on the interactions of high Mw peptides, typically molecules with Mw of over 1000 Da, have been scarce for drug-metabolizing enzymes and/or transporters. However, there are several exceptions of studies on high Mw peptides, such as cyclosporine (Mw 1202), that drive DDI via CYP3A4, P-gp, and OATP1B1 in clinical settings [29,30,31,32,33]; these studies indicate that the DDI evaluation of high Mw peptides could be indispensable.

In this study, we investigated the interaction of ATSP-7041 (Mw 1745 Da) with drug-metabolizing enzymes and transporters in vitro. We used ATSP-7041 as a model stapled peptide drug with non-natural structures. As ATSP-7041 is primarily cleared through hepatobiliary elimination [15], we focused on hepatic drug-metabolizing enzymes and transporters, namely five CYPs (CYP1A2, CYP2C9, CYP2C19, CYP3A4, and CYP2D6) and four transporters (OATP1B1, OATP1B3, P-gp, and BCRP). We first determined whether ATSP-7041 could act as a substrate or an inhibitor of these enzymes and transporters and subsequently performed detailed in vitro analysis of OATP1B1, for which ATSP-7041 could be both substrate and inhibitor; ALRN-6924, an analog of ATSP-7041, has been described as an inhibitor of OATPs [20]. In this study, we identified the DDI potential of this model peptide with cell-membrane-penetrating high Mw characteristics and the necessity for DDI studies on these types of drugs.

## 2. Materials and Methods

### 2.1. Materials

Materials for screening experiments are described in Text S1. Cyclosporine A was purchased from TCI Chemical Trading (Tokyo, Japan). Estradiol-17β-glucuronide (E_2_17βG) and estradiol-3β-glucuronide were purchased from Merck. OATP1B-expressing HEK293 cells (SB-OATP1B1-PREDICELL™-24w, SB-OATP1B1-PREDICELL™-96 w, SB-OATP1B3-PREDICELL™-24 w, and SB-OATP1B3-PREDICELL™-96 w) were purchased from KAC (Kyoto, Japan). ATSP-7041 and benzyl-ATSP-7041 were synthesized using a solid-phase peptide synthesis method with conventional coupling reagents [15]. Briefly, the peptide ATSP-7041 was synthesized using Fmoc-based solid-phase methods on NovaPEG Rink amide resin. The procedure detailing the coupling and deprotection cycles, at a 50 µmol scale, is outlined below. The NovaPEG Rink amide resin was soaked in dichloromethane for 30 min. Subsequently, a solution of Fmoc-amino acid (4 eq.), COMU (4 eq.) and DIPEA (10 eq.) dissolved in N,N-dimethylformamide (DMF) was added to the resin. Deprotection of Fmoc groups was carried out using a 20% piperidine solution in DMF. Ring closing metathesis reactions were performed using 20 mol% 2nd generation Grubbs catalyst in 1,2-dichloroethane. After the peptide synthesis, the resin was suspended in cleavage cocktail (95% trifluoroacetic acid (TFA), 2.5% water, and 2.5% triisopropylsilane) for 2 h at room temperature. The TFA solution was evaporated under a stream of N_2_, and the resulting residue was dripped into cold ether to precipitate the peptides. The crude peptides were dissolved in DMSO and purified by reversed-phase high performance liquid chromatography (HPLC) using a Inertsil WP300 C18 (5 µm, 20 mm × 250 mm) (solvent A: 0.1% TFA/water, solvent B: 0.1% TFA/acetonitrile, flow rate: 10 mL/min, gradient: 50–90% gradient of solvent B over 40 min). After purification, the peptide solutions were lyophilized. Peptide purity was assessed using analytical HPLC with an Inertsil WP300 C18 (5 µm, 4.6 mm × 250 mm) (solvent A: 0.1% TFA/water, solvent B: 0.1% TFA/acetonitrile, flow rate: 1.0 m/min, gradient: 10–90% gradient of solvent B over 30 min). Finally, the peptides were characterized by LC/mass spectrometry (LC/MS) ion trap time-of-flight (LCMS-IT-TOF, Shimadzu, Kyoto, Japan). All other reagents were commercially procured.

### 2.2. Screening Experiments of ATSP-7041 Interactions

Screening experiments of ATSP-7041 interactions are described in Text S1. Briefly, to evaluate ATSP-7041 as a substrate of CYP isoforms, we used the human liver S9 fraction and analyzed ATSP-7041 metabolism. To evaluate ATSP-7041 as an inhibitor of CYP activities, yeast microsomes expressing individual CYP and human liver S9 with individual specific substrates were used. To evaluate ATSP-7041 as a substrate of transporters, OATP1B (OATP1B1 or OATP1B3)-expressing HEK293 cells, and inside-out vesicles expressing P-gp or BCRP, were used, and ATSP-7041 uptake was analyzed. To evaluate ATSP-7041 as an inhibitor of transporter activities, the same system was used with individual proven substrates.

### 2.3. Evaluation of ATSP-7041 as a Substrate of OATP1B1

The evaluation of ATSP-7041 as a substrate for OATP1B1 was performed using OATP1B1-expressing HEK293 cells in 24- or 96-well plates according to the manufacturer’s protocol (v1.9 for OATP1B1) with minor modifications. In brief, after equilibration with the transport buffer, the buffer was replaced with a fresh transport buffer containing the indicated concentrations of ATSP-7041 or 1 μM E_2_17βG in the presence or absence of positive control inhibitors (50 μM rifampicin or 10 μM cyclosporine). After incubation for the indicated time at 37 °C, the cells were immediately rinsed twice with an ice-cold transport buffer. Substrates were extracted by incubation with 400 μL (for 24-well) or 100 μL (for 96-well) of 0.1% formic acid in acetonitrile-containing internal standard (IS) (0.125 μM benzyl-ATSP-7041 for ATSP-7041 in uptake experiment of various concentrations of ATSP-7041 or 0.0125 μM for ATSP-7041 in all other experiments of benzyl-ATSP-7041 and 0.1 μM estradiol-3β-glucuronide) for 5 min at 20–25 °C, filtered using a PVDF-based filter, diluted 1.4 times (ATSP-7041) or 2 times (E_2_17βG) with water, and analyzed using LC/MS.

### 2.4. Evaluation of ATSP-7041 as an Inhibitor of OATP1B1

The inhibitory activities of ATSP-7041 on OATP1B1 were evaluated using OATP1B-expressing HEK293 cells in 96-well plates as described above. For dose-dependent inhibition, ATSP-7041 at the indicated concentrations was added to the transport buffer, and E_2_17βG was used as the substrate. To study the pre-incubation effect, the cells were pre-incubated with 10 μM ATSP-7041 or 10 μM cyclosporine in transport buffer after equilibration with the transport buffer for 30 min at 37 °C and washed twice before the replacement of the transport buffer containing E_2_17βG.

### 2.5. LC/MS Analysis

ATSP-7041 and E_2_17βG were analyzed using LC/MS. The analysis was conducted using Ultimate3000 UHPLC with TSQ-Quantiva (Thermo Fisher Scientific, Waltham, MA, USA). A Triart Bio C18 column (3 μm, 2.1mm × 100 mm) (YMC, Kyoto, Japan) was used for LC at 50 °C. Mobile phase A consisted of 0.1% formic acid in water, and mobile phase B consisted of 0.1% formic acid in acetonitrile. The flow rate was 0.4 mL/min, and the injected sample volume was 4 μL. The gradient program of the mobile phase for each substrate is listed in Table S1. The mass spectrometer was operated in the heated electrospray ionization (ESI) mode. The parameters and mass transitions of substrates are also listed in Table S1. LC/MS-grade reagents were used for LC/MS analysis.

### 2.6. Data Analysis

LC/MS analysis data were quantified using Trace Finder 4.1 (Thermo Fisher Scientific, Waltham, MA, USA) for peak detection and area quantification. To study the transport activities of OATP1B1, the OATP1B1-mediated uptake of ATSP-7041 and E_2_17βG was determined by normalization with IS (divide the area of ion peak) and subsequent background-corrected uptake using mock cells. To evaluate the inhibitory activities of ATSP-7041 on OATP1B1, transporter activities were determined as a percent of the control (activities without ATSP-7041) at each concentration, and the half maximal inhibitory concentration (IC_50_) for the inhibition of OATP1B1 activity by ATSP-7041 was determined by fitting a curve (inhibitor concentrations vs. normalized response) using GraphPad Prism 8 (GraphPad Software, San Diego, CA, USA). To estimate Km value, an Eadie–Hofstee diagram with linear regression model was described using GraphPad Prism 9 (GraphPad Software). Differences in all figures were analyzed and all data were plotted using GraphPad Prism 9 (GraphPad Software). The results are presented as mean ± standard deviation. A one-way analysis of variance with Tukey’s multiple comparison analysis, or Welch’s *t*-test with Bonferroni’s multiple corrections, was used to determine significant differences between the groups. Results with *p* < 0.05 were considered statistically significant.

## 3. Results

### 3.1. Screening for ATSP-7041 Interactions

**Figure 1:**
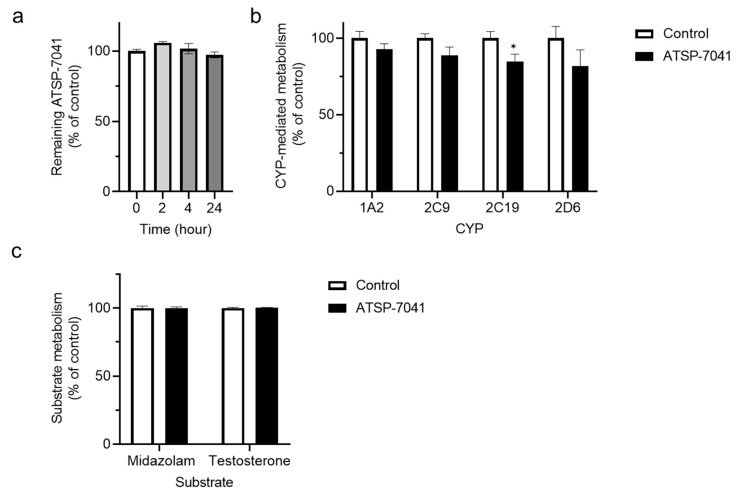
Interaction of ATSP-7041 with cytochrome P450s (CYPs). (**a**) Metabolic stability of ATSP-7041 in the human liver S9 fraction. ATSP-7041 (0.5 µM) was incubated with the human liver S9 fraction (2 mg/mL) with NADPH at 37 °C for 24 h. Values are presented as a percent of the control (time 0 h), mean ± standard deviation (SD; n = 3). (**b**) Inhibition of CYP activities by ATSP-7041. The residual activity of human CYPs with ATSP-7041 (10 μM) was determined using CYP inhibitor screening kits. After pre-incubation of ATSP-7041 with CYP enzymes, the enzymatic reaction was initiated by adding the substrate/NADP^+^ mixture and monitoring the fluorescence using a yeast microsomal preparation expressing human CYP isoforms. Data are presented as a percent of the control (without ATSP-7041), mean ± SD (n = 3). * *p* < 0.05 compared with the control. (**c**) Effects of ATSP-7041 (10 µM) on the metabolism of CYP3A4 substrates in the human liver S9 fraction. Midazolam (0.5 µM) or testosterone (5 µM) was incubated with ATSP-7041 in the human liver S9 fraction (2 mg/mL) with NADPH at 37 °C for 30 or 90 min, respectively. Data are presented as a percent of the control (without ATSP-7041), mean ± SD (n = 3).

We first determined whether ATSP-7041 could act as a substrate or an inhibitor of drug-metabolizing enzyme and transporter activities. The detailed results of the screening experiments are described in Text S2. As shown in Figure 1a, the content of ATSP-7041 (0.5 µM) was reserved over 97% of the initial time point (0 h), until up to 24 h in the human liver S9 fraction (more detailed results are shown in Text S2, and Figure S2 and Figure S3). In addition, the residual activities of CYP1A2, CYP2C9, CYP2C19, and CYP2D6 in the co-existence of ATSP-7041 (10 μM) was over 80% in yeast microsomes (Figure 1b; more detailed results are shown in Text S2, and Table S2 and Table S3), although the inhibitory effect on CYP2C19 was significant. Moreover, no significant inhibition of CYP3A4 activity was observed in the human liver S9 fraction (Figure 1c; more detailed results are shown in Text S2 and Figure S4, Figure S5, Figure S6). These results suggest a negligible or limited interaction (as a substrate or inhibitor) of ATSP-7041 with human liver CYPs in vitro.

**Figure 2:**
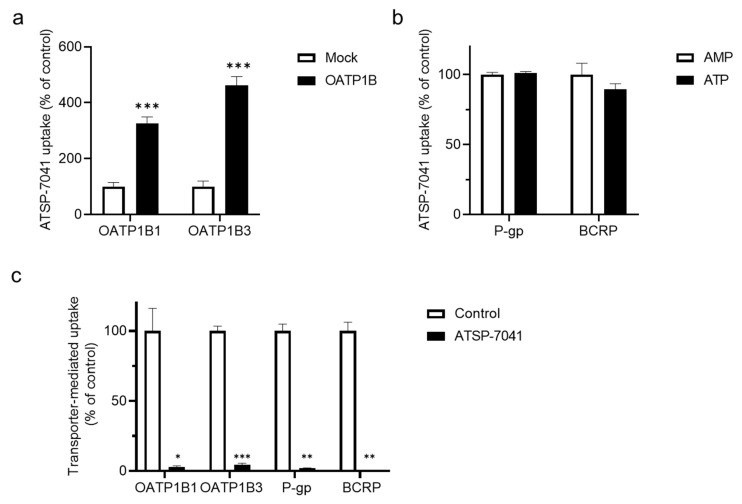
Interaction of ATSP-7041 with transporters. (**a**) Uptake of ATSP-7041 by OATP1B-expressing HEK293 cells. ATSP-7041 (0.2 μM) was incubated with OATP1B1- or OATP1B3-expressing HEK293 cells (filled square) and mock cells (open square) for 5 min at 37 °C. Data are presented as a percent of the control (mock cells), mean ± SD (n = 3). *** *p* < 0.001 compared with the control. (**b**) Uptake of ATSP-7041 by P-gp- and BCRP-expressing vesicles. ATSP-7041 (0.1 μM) was incubated with inside-out membrane vesicles expressing P-gp or BCRP with AMP (open square) or ATP (filled square) for 3 (P-gp) or 15 (BCRP) min at 37 °C. Data are presented as a percent of the control (with AMP), mean ± SD (n = 3). (**c**) Inhibition of substrate uptake of transporters by ATSP-7041. E_2_17βG was incubated as a substrate with OATP1B1- or OATP1B3-expressing HEK293 cells with (filled square) and without (open square) ATSP-7041 (10 μM) for 5 min at 37 °C. N-methyl-quinidine or lucifer yellow as a substrate was incubated with inside-out membrane vesicles expressing P-gp or BCRP with (filled square) and without (open square) ATSP-7041 (10 μM) for 3 (P-gp) or 15 (BCRP) min, respectively, at 37 °C. Transporter-mediated uptake of substrates is shown. Data are presented as a percent of the control (without ATSP-7041); mean ± SD (n = 3). * *p* < 0.05, ** *p* < 0.01, and *** *p* < 0.001, compared with the control.

Conversely, the uptake of ATSP-7041 (0.2 μM) detected in OATP1B1- and OATP1B3-expressing HEK293 cells was significant and approximately 3.3- and 4.6-fold greater than that in mock cells, respectively (Figure 2a and its control experiment are demonstrated in Text S2 and Figure S7). The evaluation of ATSP-7041, as a substrate of P-gp and BCRP, revealed that there was no significant increase in the ATP-dependent uptake of ATSP-7041 (0.1 μM) in P-gp- or BCRP-expressing vesicles (Figure 2b; more detailed results are shown in Text S2 and Figure S8). Moreover, ATSP-7041 (10 μM) showed a strong inhibition (<10% of residual transport activity) of an index substrate uptake mediated by each transporter analyzed in this study (Figure 2c). These screening results show that ATSP-7041 could act as a substrate as well as an inhibitor of OATP activities and as an inhibitor of P-gp and BCRP activities. As ALRN-6924, an analog of ATSP-7041, is reported as an inhibitor of OATP activities [20] and a high frequency of DDI was observed via OATP1B1 over OATP1B3 [25], we subsequently performed a detailed in vitro analysis with OATP1B1.

### 3.2. Characterization of ATSP-7041 as a Substrate of OATP1B1

**Figure 3:**
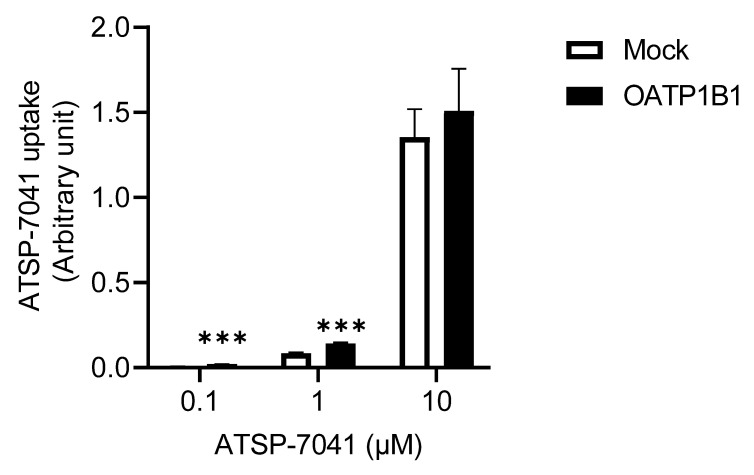
Uptake of ATSP-7041 at various concentrations by OATP1B1-expressing HEK293 cells. ATSP-7041 (0.1, 1, and 10 μM) was incubated with OATP1B1-expressing HEK293 cells (filled square) and mock cells (open square) for 5 min at 37 °C. Data are presented as a percent of the control (mock cells); mean ± SD (n = 3). *** *p* < 0.001, compared with the control.

To further characterize ATSP-7041 as a substrate of OATP1B1, we first examined the dose-dependent changes in the uptake of ATSP-7041 by OATP1B1. Figure 3 shows a substantial OATP1B1-dependent uptake at 0.1 μM (3.4-fold) and 1 μM (1.7-fold), although this uptake was saturated at 10 μM (1.1-fold; not significant). In addition, although it was described with three points of concentrations, the K_m_ value estimated using the Eadie–Hofstee diagram was 0.85 μM.

**Figure 4:**
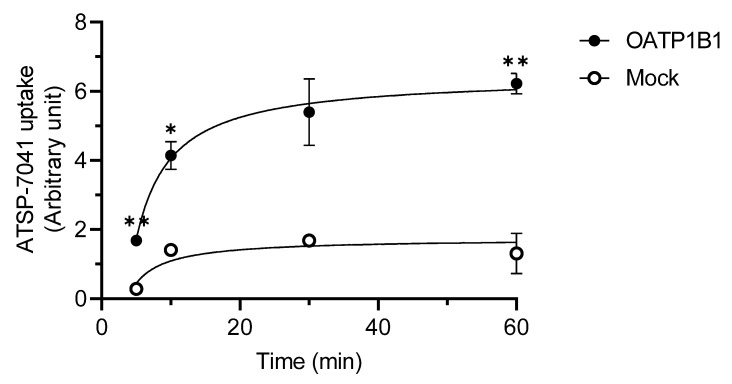
Time-dependent uptake of ATSP-7041 by OATP1B1-expressing HEK293 cells. ATSP-7041 (0.2 μM) was incubated with OATP1B1-expressing HEK293 cells (filled circle) and mock cells (open circle) for indicated periods at 37 °C. Data are presented as mean ± SD (n = 3). * *p* < 0.05 and ** *p* < 0.01, compared with the control (mock cells).

Next, we examined the time-dependent changes in the uptake of ATSP-7041 by OATP1B1. As shown in Figure 4, the OATP1B1-dependent uptake of ATSP-7041 (0.2 μM) was observed at all studied time points, although this uptake was not significant at 30 min. In addition, the OATP1B1-dependent uptake reached a steady state before 1 h.

**Figure 5:**
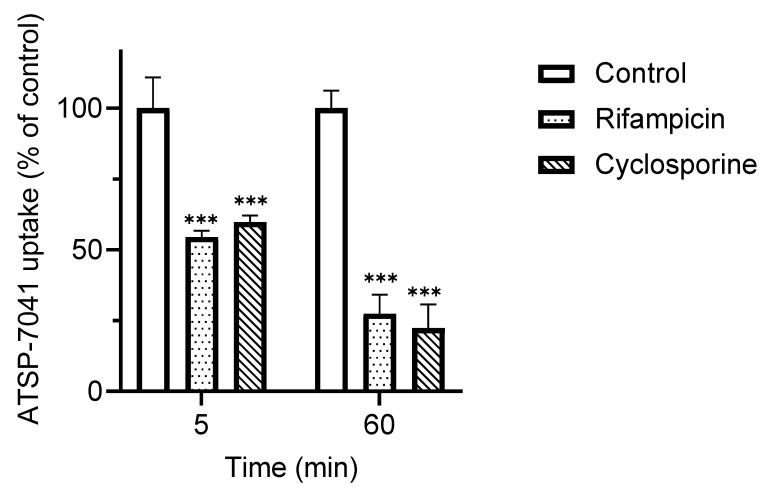
Effects of known inhibitors on ATSP-7041 uptake by OATP1B1-expressing HEK293 cells. ATSP-7041 (0.2 μM) was incubated with OATP1B1-expressing HEK293 cells and mock cells with an inhibitor, rifampicin (50 μM), or cyclosporine (10 μM), for 5 or 60 min at 37 °C. OATP1B1-mediated uptake of ATSP-7041 is shown. Data are presented as a percent of the control (without inhibitors), mean ± SD (n = 3). *** *p* < 0.001, compared with the control.

We further examined the effect of the index inhibitors of OATP1B1 activity, cyclosporine, and rifampicin, on the uptake of ATSP-7041. As shown in Figure 5, the OAT1B1-dependent uptake of ATSP-7041, at both 5 and 60 min, was significantly inhibited by both cyclosporine and rifampicin. Under the same experimental conditions, with 5 min co-incubation, the uptake of the control substrate E_2_17βG by OATP1B1-expressing HEK293 cells was inhibited by rifampicin (<4% without inhibitor) (Figure S1).

### 3.3. Characterization of ATSP-7041 as an Inhibitor of OATP1B1 Activity

**Figure 6:**
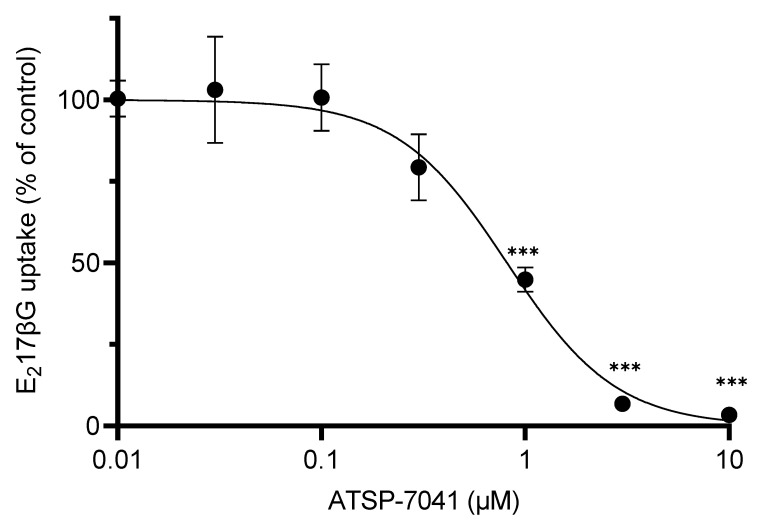
Inhibition of transporter activities of OATP1B1 by ATSP-7041. E_2_17βG was incubated as a substrate with OATP1B1-expressing HEK293 cells and mock cells with various concentrations of ATSP-7041 for 5 min at 37 °C. OATP1B1-mediated uptake of the substrate is shown. Values plotted as a percent of the control (without ATSP-7041), mean ± SD (n = 3). *** *p* < 0.001, compared with the control. The half maximal inhibitory concentration (IC_50_) value, with 95% confidence interval, for ATSP-7041 was 0.81 µM (0.66–1.00).

Along with the characterization of ATSP-7041 as a substrate, we characterized ATSP-7041 as an inhibitor of OATP1B1 activity. As shown in Figure 6, ATSP-7041 showed a dose-dependent inhibition of the substrate E_2_17βG’s uptake, mediated by OATP1B1. A significant inhibition of ATSP-7041 activity was observed from 1 μM and >96% of inhibition was observed at 10 μM. In addition, the IC_50_ value of ATSP-7041 for OATP1B1 activity inhibition was 0.81 μM (95% confidence interval: 0.66–1.00 μM).

**Figure 7:**
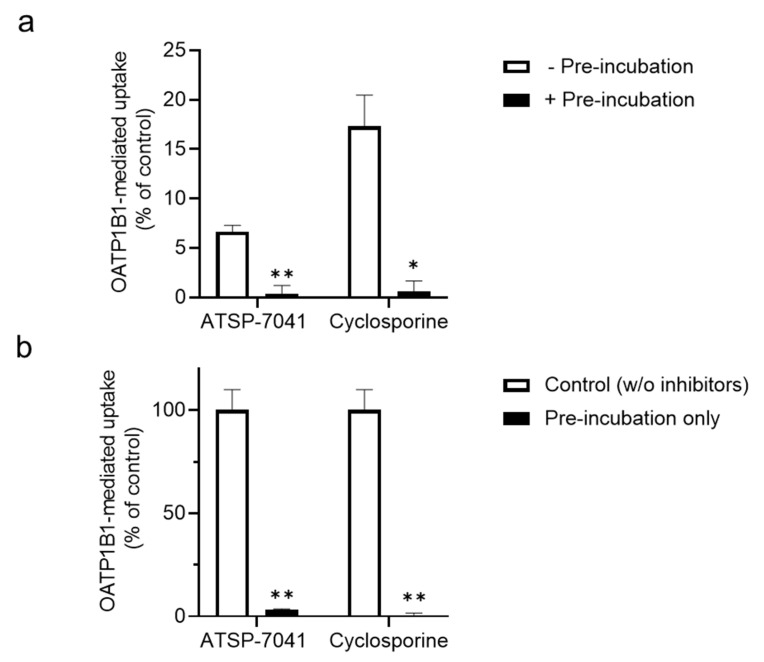
Effects of pre-incubation on inhibition of substrate uptake by ATSP-7041 in OATP1B1-expressing HEK293 cells. (**a**) Effects of additional pre-incubation with ATSP-7041 on substrate uptake. E_2_17βG was incubated as a substrate with OATP1B1-expressing HEK293 cells or mock cells with ATSP-7041 (10 μM) or cyclosporine (10 μM) for 5 min at 37 °C, with (filled square) or without (open square) additional pre-incubation for 30 min at 37 °C. OATP1B1-mediated uptake of the substrate is shown. Data are presented as a percent of the control (without inhibitors), mean ± SD (n = 3). * *p* < 0.05 and ** *p* < 0.01, compared between with and without pre-incubation. (**b**) Effects of pre-treatment of OATP1B1 with ATSP-7041 on the following substrate uptake. ATSP-7041 (10 μM) or cyclosporine (10 μM) was pre-incubated with OATP1B1-expressing HEK293 cells or mock cells for 30 min at 37 °C; after washing the cells, E_2_17βG was incubated as a substrate in the absence of ATSP-7041 or cyclosporine. OATP1B1-mediated uptake of the substrate is shown. Data are presented as a percent of the control (without inhibitors), mean ± SD (n = 3). ** *p* < 0.01 compared with the control.

As the peptide-type index inhibitor cyclosporine shows the enhanced inhibition of OATP1B1 activity by pre-incubation and inhibition following removal [34], we further examined whether ATSP-7041 exerts a similar inhibitory effect on OATP1B1. As shown in Figure 7a, the pre-incubation of cells with ATSP-7041 further enhanced the inhibition of OATP1B1 activity, similar to the effect of cyclosporine. In addition, even in the absence of ATSP-7041 during the uptake of the OATP1B1 substrate, the uptake of the OATP1B1 substrate E_2_17βG was substantially inhibited by pre-incubation of ATSP-7041, similar to cyclosporine (Figure 7b).

## 4. Discussion

In this study, we evaluated the interactions of ATSP-7041, as a model stapled peptide of high Mw with cellular-membrane-penetrating characteristics (also with non-natural structures), with hepatic drug-metabolizing enzymes and transporters, as the biliary clearance of ATSP-7041 has been reported [15]. Our screening results demonstrated the following. Firstly, there is negligible or limited interaction of ATSP-7041 with human liver CYPs in vitro. Secondly, ATSP-7041 could act as both a substrate and inhibitor of OATP activities in vitro, and an inhibitor of P-gp and BCRP activities. Our results suggest that ATSP-7041 has the potential for interaction with hepatic transporters.

Further analysis of OATP1B1 revealed the following. Firstly, an OATP1B1-dependent uptake of ATSP-7041 was observed with an estimated K_m_ value of 0.85 μM (Figure 3), which is within the range of the K_m_ values of other clinically used substrate drugs of OATP1B1 with a low Mw [35]. In addition, the time-dependent profile of OATP1B1-dependent uptake reached the steady state before 60 min, similar to that of typical substrates [35,36,37]. Moreover, the OATP1B1-dependent uptake of ATSP-7041 was inhibited by typical inhibitors (Figure 5). Collectively, ATSP-7041 (Mw 1745 Da) might be an OATP1B1 substrate. Reports on OATP1B1 substrates have almost been limited to small molecules of Mw < 1000 Da [36,38]. At a Mw of over 1000 Da, only cyclosporine (Mw 1202 Da) for OATP1B1/1B3, caspofungin (Mw 1093 Da) for OATP1B1, and CCK-8 (Mw 1141 Da) for OATP1B3 are characterized as substrates; molecules exceeding this size are typically not considered OATP1B1 substrates. However, our results demonstrated that molecules, even over 1500 Mw, could be a substrate for OATP1B1 and may cause DDI, in combination with its typical inhibitors.

It remains unclear whether ATSP-7041 can act as an OATP1B1 substrate in vivo. However, OATP1B1 is expressed on the sinusoidal membrane of hepatocytes and can facilitate the liver uptake of its substrate anionic drugs [38]. A previous study on the tissue distribution of ATSP7041 has demonstrated its hepatobiliary excretion in mice, although its mechanism is not delineated [15]. Our results demonstrated that the estimated K_m_ value of ATSP-7041 is compatible with the K_m_ value of substrate drugs of OATP1B1 defined in vivo [35]. Thus, ATSP-7041 may act as an OATP1B1 substrate in vivo, and OATP1B1-mediated uptake, in addition to passive cellular penetration, could be a feasible mechanism contributing to the hepatobiliary excretion of ATSP-7041. In addition, OATP1B1 uptake reached a steady state before 1 h in our study (Figure 4), whereas cellular transparency was determined at 4 h in studies that analyzed cellular penetration of ATSP-7041 [15,16,17]. The differences in time scale between OATP1B1-mediated uptake and cellular penetration could support the idea of the selected uptake of ATSP7041 by OATP1B1 in the liver. Future in vivo validation with typical inhibitors, and knock-in/knock-out animal models of OATP1B1, could address the involvement of OATP1B1 in the hepatic uptake of ATSP-7041.

Along with the characterization of ATSP-7041 as an OATP1B1 substrate, we demonstrated that ATSP-7041 inhibits OATP1B1 activity in a concentration-dependent manner, with an IC_50_ value of 0.81 μM (Figure 6). Cyclosporine A, a well-known strong inhibitor of OATP1B1 activity, causes clinical DDI, owing to the inhibition of the OATP1B1-mediated uptake of co-administrated substrate drugs, such as cerivastatin and pitavastatin [29,39]. Cyclosporine A inhibits OATP1B1 activity in vitro with an IC_50_ of 0.31–2.2 μM [30,37,40,41]. Thus, the IC_50_ for OATP1B1 by ATSP-7041 observed in our in vitro study was comparable to that of cyclosporine A. In addition, the long-lasting inhibition of OATP1B1 activity by ATSP-7041 was observed in the present study (Figure 7). Therefore, ATSP-7041 may act as an inhibitor of OATP1B1 activity in clinical settings, similar to cyclosporine A, although pharmacokinetic parameters, such as the plasma-binding ratio and C_max_ of the specified clinical dose, should be evaluated to predict the risk of clinical DDI. In a phase I study of ALRN-6924, an analog of ATSP-7041 [20], the maximum C_max_ was 50 μM; thus, the risk of DDI of ATSP-7041 cannot be excluded when assuming an unbound rate at 1%.

In clinical settings, ATSP-7041 is used for cancer therapy because ATSP-7041 suppresses tumor growth by activating the p53 pathway [10]. As combination therapy with anticancer drugs has been developed to maximize the efficacy of tumor therapy [42,43], ATSP-7041 could be used in combination therapy against cancer. Various cancer drugs have been reported to be substrates or inhibitors of OATP1B1 activity. For example, methotrexate [31], gimatecan, flavopiridol, and rapamycin [44] are substrates of OATP1B1, and ATSP-7041 may increase their plasma concentration by inhibition of OATP1B1. On the other hand, tyrosine kinase inhibitors such as axitinib and sorafenib [45] are inhibitors of OATP1B1 activity, and the plasma concentration of ATSP-7041 may be increased by them. Therefore, when co-administered with these cancer drugs, the DDI risk of ATSP-7041 should be carefully evaluated in clinical settings. In addition, numerous patients with cancer have multiple comorbidities, such as hypertension, diabetes, and cardiovascular disease [46], and this has led to combination therapy with anticancer and therapeutic drugs for these comorbidities. Similarly, caution should be exercised when evaluating the DDI risk of ATSP-7041 if ATSP-7041 is co-administered with these drugs, which are also substrates or inhibitors of OATP1B1 activity.

The present study has demonstrated that ATSP-7041 exhibits the characteristics of an OATP1B1 substrate and is a strong inhibitor of OATP1B1. Consequently, ATSP-7041 has the potential to competitively inhibit the transport of other molecules by OATP1B1, and this inhibitory effect is likely reversible. However, our study also demonstrated that ATSP-7041, similar to cyclosporine, exerts a long-lasting inhibition of OATP1B1 activity. Although the exact mechanism responsible for this long-lasting inhibition remains unclear, it is evident that the recovery from ATSP-7041 inhibition takes a longer time. Furthermore, since ATSP-7041 is an OATP1B1 substrate, its inhibitory activity may be related to its affinity to OATP1B1. To date, apart from our study, there have been no reports characterizing a peptide with a molecular weight exceeding 1500 Mw as a substrate of OATP1B1. Thus, future studies should be focused on the accumulation of practical data and the exploration of the structure–activity relationships of peptides (including linear peptides, in addition to stapled peptides) with molecular weights exceeding 1500 Mw, particularly regarding their affinity as OATP1B1 substrates.

In the present study, ATSP-7041 was characterized as an inhibitor of not only OATP1B1 activity, but also OATP1B3, P-gp, and BCRP activities through screening experiments (Figure 2). As OATP1B3, P-gp, and BCRP are known to show clinical DDIs [25], the DDI risk of ATSP-7041 should be evaluated carefully in clinical settings when co-administered with drugs of these substrates. In vivo studies are warranted to evaluate the risk of activity inhibition of these transporters by ATSP-7041 in clinical settings.

In conclusion, our study suggests that ATSP-7041 is not only a substrate but also a strong inhibitor of OATP1B1 activity. In addition, it demonstrated that ATSP-7041 could act as a strong inhibitor of OATP1B3, P-gp, and BCRP activities. Therefore, careful evaluation of ATSP-7041 is required for risk prediction of clinically relevant DDIs. As ATSP-7041 is a stapled α-helical peptide with non-natural structures, which is expected to be a new therapeutic modality, DDI studies are necessary, especially with transporters, in the development of these cellular-membrane-penetrating peptide drugs, despite their high Mw, according to the issued DDI guidelines including ICH M12 draft.

## Data Availability

All datasets are provided as Supplementary Tables.

## Supplementary Materials

The following supporting information can be downloaded at: https://www.mdpi.com/article/10.3390/biom13061002/s1, Text S1: Supplementary Materials and Methods; Text S2: Supplementary results; Figure S1: OATP1B1-mediated uptake of E_2_17βG and its inhibition by rifampicin; Figure S2: Metabolic stability of ATSP-7041 in human liver S9 fraction; Figure S3: Vender differences in human liver S9 on metabolic stability of ATSP-7041; Figure S4: Time course in metabolism of midazolam and testosterone in human liver S9 fraction; Figure S5: Effects of ATSP-7041 on the metabolism of CYP3A4 substrates in human liver S9 fraction. Figure S6: Vender differences in human liver S9 fraction in terms of effects of ATSP-7041 on metabolism of CYP3A4 substrates; Figure S7: OATP1B-mediated uptake of E_2_17βG; Figure S8: P-gp- and BCRP-mediated uptake of ATSP-7041; Table S1: Parameters used in LC/MS analysis; Table S2: Substrates and positive control inhibitors used for CYPs inhibition screening; Table S3: Inhibitory activities of ATSP-7041 on CYPs in yeast microsomes.
