# Supplementary material for: Identification of the Stapled α-Helical Peptide ATSP-7041 as a Substrate and Strong Inhibitor of OATP1B1 In Vitro
Source: Biomolecules. 2023 Jun 16;13(6):1002. doi: 10.3390/biom13061002 (PMC10296347; doi:10.3390/biom13061002)
Supplement: Supplementary file 1 [file biomolecules-13-01002-s001.zip › biomolecules-2434245-supplementary.pdf]

Text S1. Supplementary Materials and Methods.

1. Materials

NADPH generation system and cyclosporine D were purchased from Funakoshi Co., Ltd. (Kyoto, Japan). Human liver S9 was purchased from Corning (A; Ultrapool Human Liver S9, 150-Donor Pool; Corning, NY, USA), Thermo Fisher Scientific Inc. (B; Human S9 fractions; Waltham, MA, USA), Merck (C; S9 from Liver, Pooled; Darmstadt, Germany), and Veritas (D; Male Human Liver Pooled S9; Tokyo, Japan). Midazolam, midazolam-d4, 1′-hydroxymidazolam, testosterone, testosterone-d3, and 6β-hydroxytestosterone, were purchased from Merck. Cytochrome P450 (CYPs) inhibitor screening kits (Cytochrome P450 1A2 Inhibitor Screening Kit (Fluorometric), Cytochrome P450 2C9 Inhibitor Screening Kit (Fluorometric), Cytochrome P450 2C19 Inhibitor Screening Kit (Fluorometric), Cytochrome P450 2D6 Inhibitor Screening Kit (Fluorometric), and Cytochrome P450 3A4 Inhibitor Screening Kit (Fluorometric)) were purchased from BioVision. Inc. (Milpitas, CA, USA). Reversed vesicles (HEK293-Mock-CTRL, BCRP-HEK293, and MDR1-HEK293) and reagents (PREDIVEZ™Reagent Kit for MDR1/P-gp and PREDIVEZ™Reagent Kit for BCRP) for vesicle assay of P-gp and BCRP, respectively, were purchased from Merck. All other reagents were commercially available.

2. Evaluation of ATSP-7041 as a substrate of CYPs

The metabolism of ATSP-7041 or cyclosporine A by CYPs was evaluated in human liver S9, with or without the NADPH generation system. S9 (A), at a 2 mg/mL protein concentration in 100 mM phosphate buffer (pH 7.4) containing 3 mM $MgCl_2$, was pre-incubated for 5 min at 37 °C, and the reaction was initiated by the addition of the NADPH generation system and ATSP-7041 (0.5 μM) or cyclosporine A (0.5 μM). The reaction solution (400 μL) was then incubated at 37 °C. At 0, 2, 4, and 24 h, 50 μL of the reaction solution was collected, and the reaction was terminated by adding 150 μL of cold 1.3% formic acid in acetonitrile containing internal standard (IS) (0.3 μg/mL benzyl-ATSP-7041 and 0.2 μg/mL cyclosporine D). After vortexing and centrifugation at 20,000 ×g for 10 min at 4 °C, the supernatant was filtered using a polyvinylidene difluoride (PVDF)-based filter (Ultrafree-MC; pore size: 0.22 μm; Millipore Sigma; St. Louis, MO, USA), diluted 1.25 times with water, and then analyzed by LC/mass spectrometry (LC/MS). To evaluate vendor differences in human liver S9, the reactions were also conducted for 24 h with human liver S9 (B, C, and D).

3. Evaluation of ATSP-7041 as an inhibitor of CYPs

The inhibitory activity of ATSP-7041 on CYPs was first evaluated using a yeast microsomal preparation expressing human CYPs (CYPs Inhibitor Screening Kits) according to the manufacturer's protocol. In brief, after pre-incubation (37 °C, 15 min) of ATSP-7041 (10 μM in reaction composition) with CYP enzymes in the assay buffer (70 μL), the reaction was initiated by adding 30 μL of the substrate/$NADP^+$ mixture and monitoring the fluorescence for the indicated periods of times. The substrates, positive control inhibitors, and fluorescence measurement conditions are listed in Table S2.

Then, the inhibitory activity of ATSP-7041 on the metabolism of CYP3A4 substrates in human liver S9 was precisely evaluated using midazolam and testosterone as its typical substrates. The same reaction composition of human liver S9, except for the substrates, was used, as described above. The inhibitory activity of ATSP-7041 (10 μM) on the metabolism of midazolam (0.5 μM) was evaluated by measuring midazolam and 1′-hydroxymidazolam 30 min

after the time course assessment (0, 5, 10, 20, 30, 40, and 60 min). Similarly, the inhibitory activity of ATSP-7041(10 μM) on the metabolism of testosterone (0.5 μM) was evaluated by measuring testosterone and 6β-hydroxytestosterone at 90 min, after the time course assessment (0, 10, 20, 40, 60, 90, and 120 min). Other human liver S9 (B, C, and D) fractions were also used to evaluate vendor differences. Midazolam, 1′-hydroxymidazolam, testosterone, and 6β-hydroxytestosterone were detected using LC/MS.

4. Evaluation of ATSP-7041 as a substrate of transporters

The evaluation of ATSP-7041 as a substrate for OATP1B1 and OATP1B3 transporters was performed using OATP1B-expressing HEK293 cells in 24-well plates according to the manufacturer's protocol (v1.9 for OATP1B1 and v2.0 for OATP1B3) with minor modifications. In brief, after equilibration with the transport buffer, the buffer was replaced with a fresh transport buffer containing ATSP-7041 (0.2 μM) or $E_2$17βG (1 μM for OATP1B1 or 3 μM for OATP1B3). After incubation for 5 min at 37 °C, the cells were immediately rinsed twice with ice-cold transport buffer. Substrates were extracted by incubation with 400 μL of 0.1% formic acid in acetonitrile containing IS (0.0125 μM benzyl-ATSP-7041 and 0.1 μM estradiol-3β-glucuronide) for 5 min at 20-25 °C, filtered using a PVDF-based filter, diluted 1.4 times (ATSP-7041) or 2 times ($E_2$17βG) with water, and analyzed by LC/MS.

The evaluation of ATSP-7041 as a substrate for P-gp and BCRP transporters was performed using inside-out vesicles and reagents for the vesicle assay, according to the manufacturer's protocol (Version 1.1 for MDR1 and 1.3 for BCRP), with minor modifications. Briefly, ATSP-7041 (0.1 μM) was pre-incubated for 15 min at 37 °C in a 96-well plate containing the transporter vesicles (25 μg protein/well). The reaction was initiated by adding 4 mM ATP or AMP and incubating for 3 (P-gp) or 15 min (BCRP) at 37 °C. The reaction was stopped by the addition of ice-cold washing buffer and transferred to a filter plate. The liquid was removed under vacuum and washed with a washing buffer. After drying the filter plate, the substrates were extracted with 300 μL of 0.1% formic acid in 80% methanol containing IS (0.0125 μM benzyl-ATSP-7041), filtered using a PVDF-based filter, and analyzed by LC/MS. The assay was performed with the same experimental procedure using a positive control for P-gp, 2 μM N-methyl-quinidine (NMQ) as substrate and 25 ng/mL quinidine as IS. For BCRP, 50 μM Lucifer yellow as substrate was used with same experimental procedure to extract vesicles. Lucifer yellow uptake was assessed using 150 μL of the detector solution and analyzed using fluorescence spectroscopy (Ex/Em = 435/530 nm). The unspecified binding of substrates to vesicles was evaluated using vesicles from mock cells in the same plate.

5. Evaluation of ATSP-7041 as an inhibitor of transporters

The inhibitory activities of ATSP-7041 on OATP1B1 and OATP1B3 were evaluated using OATP1B-expressing HEK293 cells in 96-well plates as described above. The volume of substrate extraction was 100 μL owing to the use of a 96-well plate. ATSP-7041 (10 μM) was added to the transport buffer and $E_2$17βG was used as the substrate, and 50 μM rifampicin was used as a positive control inhibitor.

The inhibitory activities of ATSP-7041 on P-gp and BCRP were evaluated using vesicles and reagents for the vesicle assay, as described above. ATSP-7041 (10 μM) was added to the reaction solution with NMQ (P-gp) or Lucifer yellow (BCRP) as substrates, and 100 μM verapamil (P-gp) or 300 μM omeprazole (BCRP) was used as a positive control inhibitor.

6. LC/MS analysis

ATSP-7041, cyclosporine A, midazolam, 1′-hydroxymidazolam, testosterone, 6β-hydroxytestosterone, NMQ, and $E_2$17βG were analyzed by LC/MS. LC/MS analysis was conducted using an Ultimate3000 UHPLC with TSQ-Quantiva (Thermo Fisher Scientific, Waltham, MA). A Triart Bio C18 column (3 μm, 2.1 × 100 mm) (YMC, Kyoto, Japan) was used for LC at 50 °C. Mobile phase A consisted of 0.1% formic acid in water, and mobile phase B consisted of 0.1% formic acid in acetonitrile. The flow rate was 0.4 mL/min, and the injected sample volume was 4 μL. The gradient program of the mobile phase for each substrate and metabolite is listed in Table S1. The mass spectrometer was operated in heated electrospray ionization (ESI) mode. The parameters and mass transitions of substrates and metabolites are listed in Table S1. LC-MS-grade reagents were used for LC-MS analysis.

7. Data analysis

Quantification of LC/MS analysis was performed using Trace Finder 4.1 (Thermo Fisher Scientific, Waltham, MA, USA) for peak detection and area quantification. For transporter activities of OATP1B1 and OATP1B3, uptake of $E_2$17βG was determined by background-corrected uptake using mock cells. For transporter activities of P-gp and BCRP, ATP-dependent transport was determined by subtracting the values in the presence of AMP from the values in the presence of ATP. To evaluate the inhibitory activities of ATSP-7041 on transporters, transporter activities were determined as the percentage of control (activities without ATSP-7041).

Text S2. Supplementary results.

1. Evaluation of ATSP-7041 as a substrate of CYPs

First, to evaluate ATSP-7041 as a substrate of CYPs, the metabolism of ATSP-7041 by CYPs was assessed in the human liver S9. The content of ATSP-7041 (0.5 µM) was not significantly different between the samples in the presence and absence of NADPH at 24 h and remained at 91.6% of the control (time 0 h, without NADPH) after 24 h of reaction with NADPH (Figure S2A). Under the same experimental conditions, the substrate of CYPs, cyclosporine A (0.5 µM), diminished to 7.3% of the initial content after a 24-h reaction with NADPH (Figure S2B). In addition, ATSP-7041 was stable with human liver S9 from three different vendors (B, C, and D) (Figure S3).

2. Evaluation of ATSP-7041 as an inhibitor of CYPs

2.1. Inhibition of CYP activities by ATSP-7041 in yeast microsomes

Next, we evaluated the inhibitory activities of ATSP-7041 on five major hepatic human CYPs using yeast microsomes-based CYP inhibitor screening kits. As shown in Figure 1B and Table S3, weak inhibition (residual enzyme activity, 68.3% of control) of CYP3A4 by ATSP-7041 (10 μM) was observed, whereas other CYPs, 1A2, 2C9, 2C19, and 2D6 showed residual activities within 81.8 to 92.6% of the control in the presence of ATSP-7041 (10 μM). Under the same experimental conditions for each CYP, strong inhibition by positive control inhibitors was confirmed, as shown in Table S3.

2.2. Inhibitory activities of ATSP-7041 on the metabolism of CYP3A4 substrates in S9

Because ATSP-7041 showed a weak inhibition of CYP3A4 activity by the inhibitor screening kits, we evaluated the inhibitory activities of ATSP-7041 on CYP3A4 in human liver

S9 using the typical CYP3A4 substrates, midazolam and testosterone. As shown in Figure S4A and B, levels of midazolam were diminished and those of its metabolite, 1′-hydroxymidazolam, were increased after 30 min of reaction with NADPH. Similarly, testosterone levels were diminished and those of its metabolite, 6β-hydroxytestosterone, were increased after 90 min of reaction with NADPH (Figure S4C and D). However, there was no inhibition of midazolam or testosterone metabolism by ATSP-7041 (10 μM) in human liver S9 (Figure 1C and Figure S5). In addition, ATSP-7041 showed no significant inhibition of midazolam and testosterone metabolism in human liver S9 from three different vendors (B, C, and D) (Figure S6).

3. Evaluation of ATSP-7041 as a substrate of transporters

We evaluated ATSP-7041 as a substrate of OATPs in transporter-expressing HEK293 cells. As shown in Figure 2A, the uptake of ATSP-7041 (0.2 μM) detected in OATP1B1- and OATP1B3-transfected HEK293 cells was approximately 3.3- and 4.6-fold greater than that in mock cells, respectively. Under the same experimental conditions, the uptake of a control substrate, $E_2$17βG, by OATP1B1- and OATP1B3-transfected HEK293 cells was approximately 611- and 137-fold greater than that by mock cells, respectively (Figure 7).

On the other hand, evaluation of ATSP-7041 as a substrate of the two ABC transporters was conducted using a vesicle assay with reversed membrane vesicles. As shown in Figure 2B and Figure S8A and B, there was no significant increase in the ATP-dependent uptake of ATSP-7041 (0.1 μM) in P-gp- or BCRP-expressing vesicles, and these were not significantly different from that of mock vesicle controls. Under the same experimental conditions, ATP-dependent uptake of the positive control substrate NMQ by P-gp or of Lucifer yellow by BCRP was clearly observed compared to that of mock vesicles (Figure S8C and D).

4. Evaluation of ATSP-7041 as an inhibitor of transporters

The inhibitory activities of ATSP-7041 were evaluated by the same system used for the evaluation of ATSP-7041 as a substrate with known substrates ($E_2$17βG for OATP1B1 and OATP1B3, NMQ for P-gp, and Lucifer yellow for BCRP, respectively). ATSP-7041 showed inhibition of the substrate uptake mediated by OATP1B1, OATP1B3, P-gp, and BCRP (Figure 2C).

Table S1. Parameters used in LC/MS analysis.

Analyte	Transition	Polarity	Ion source voltage	Source temp.	Collision energy	LC gradient program
ATSP-7041	872.93/864.43	Positive	4000	Ion transfer tube: 350, Vaporizer: 200	14	40% B (0-1 min) - 90% B (7 min) - 95% B (7.1-10 min) - 40% B (10.1-12 min)
Benzyl-ATSP-7041 (IS)	903.95/895.40				12	
Cyclosporine A	1202.8/1184.8	Positive	4000		25	40% B (0-1 min) - 90% B (7 min) - 95% B (7.1-10 min) - 40% B (10.1-12 min)
Cyclosporine D (IS)	1216.9/1198.9				25	
Midazolam	326.10/291.16	Positive	3500		35	20% B (0-1 min) - 80% B (3 min) - 95% B (3.1-5 min) - 20% B (5.1-7 min)
1'-hydroxymidazolam	342.10/324.11				20	
Midazolam-d4 (IS)	330.12/295.18				25	
Testosterone	289.32/97.2	Positive	3500		30	10% B (0-1 min) - 80% B (3 min) - 95% B (3.1-5 min) - 10% B (5.1-7 min)
6β-hydroxytestosterone	305.35/269.25				20	
Testosterone-d3 (IS)	292.34/97.22				25	
Estradiol-17β-glucuronide	447.2/271.17	Negative	2500		40	20% B (0-1 min) - 40% B (5 min) - 95% B (5.1-8 min) - 20% B (8.1-10 min)
Estradiol-3β-gluclonide (IS)	447.2/271.17				30	
N-methyl-quinidine	339.24/339.25	Positive	3500		20	5% B (0-1 min) - 45% B (3 min) - 95% B (3.1-5 min) - 5% B (5.1-7 min)
Quinidine (IS)	325.19/184.1, 253.2, 307.2				30	

Table S2. Substrates and positive control inhibitors used for CYPs inhibition screening.

CYP isoform	Substrate	Concentration	Positive control inhibitor	Concentration	Detection	Kit No.
1A2	3-cyano-7-hydroxycoumarin (3-CHC)	4 μM	α-naphthoflavone	6 μM	Ex/Em = 405/480	K894
2C9	7-hydroxy-4-(trifluoromethyl)-coumarin (7-HFC)	40 μM	Sulfaphenazole	60 μM	Ex/Em = 405/495	K896
2C19	3-cyano-7-hydroxycoumarin (3-CHC)	6 μM	Ticlopidine	30 μM	Ex/Em = 405/480	K849
2D6	3-[2-(N,N-diethyl-N-methylammonium)ethyl]-7-hydroxy-4-methylcoumarin (AHMC)	4 μM	Quinidine	3 μM	Ex/Em = 390/480	K704-200
3A4	Resorufin	2 μM	Ketoconazole	30 μM	Ex/Em = 530/580	K702-200

Table S3. Inhibitory activities of ATSP-7041 on CYPs in yeast microsomes.

CYPs isoform	Residual activity (% of control)	
	ATSP-7041 (10 μM)	Positive control inhibitor
1A2	92.6 ± 3.62	≈ 0 ***
2C9	88.8 ± 5.31	8.59 ± 2.06***
2C19	84.5 ± 4.96*	3.12 ± 0.28***
2D6	81.8 ± 10.6	2.25 ± 0.24***
3A4	68.3 ± 2.49	2.32 ± 0.16***

Residual activity (% of control, i.e. without test chemical for inhibition) of human CYPs with ATSP-7041 (10 μM) in CYPs inhibition screening. The activity of CYPs were calculated as background-corrected reaction rates using the linear phase of reaction progress curve. The positive control inhibitors and their concentrations were listed in Table S1. Data are presented as the mean ± S.D. (n = 3). * $p < 0.05$, *** $p < 0.001$ compared with control (without inhibitor).

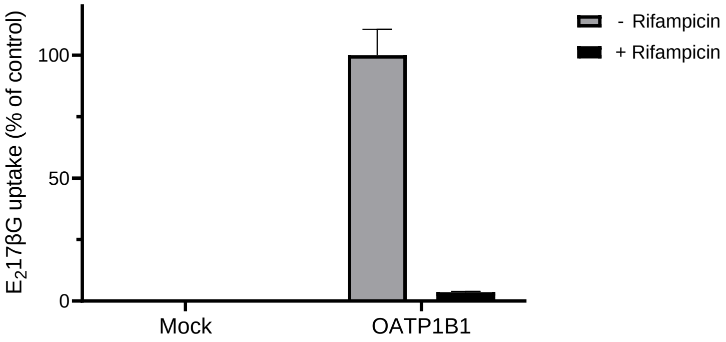

Figure S1. OATP1B1-mediated uptake of E217βG and its inhibition by rifampicin.
Uptake of E217βG (1 μM) by OATP1B1-expressing HEK293 cells and mock cells with (-■-) or without (-■-) rifampicin (50 μM). Data are presented as % of control (OATP1B1, without Rifampicin), the mean ± S.D. (n = 3).

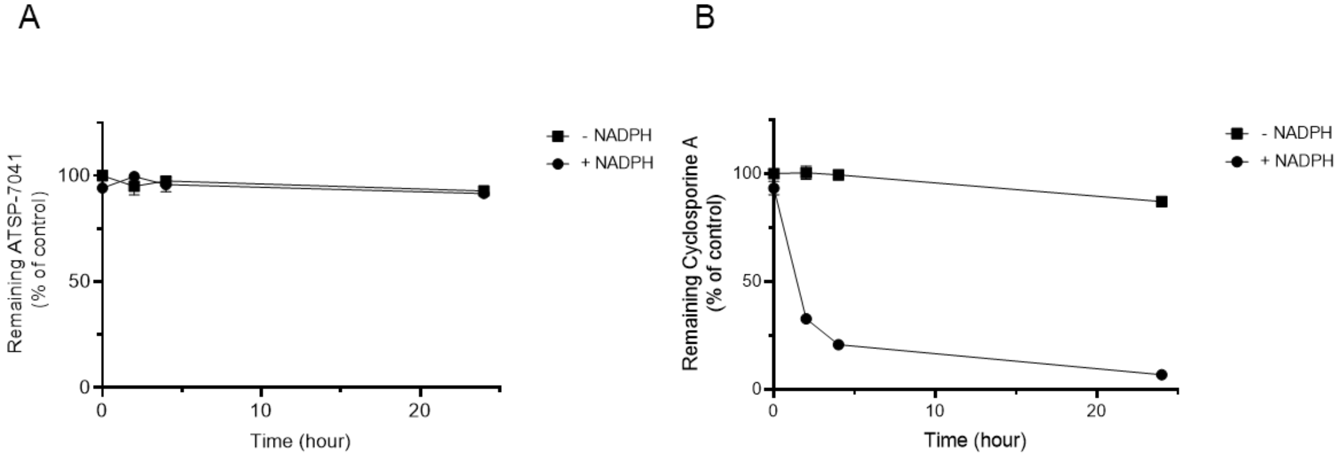

Figure S2. Metabolic stability of ATSP-7041 in human liver S9 fraction.
ATSP-7041 (0.5 μM) (A) and cyclosporine A (0.5 μM) (B) were incubated with human liver S9 fraction (2 mg/mL, vender A) with (-●-) or without (-■-) NADPH at 37 °C. Values are plotted as % of control (without NADPH, time 0 h), mean ± S.D. (n = 3).

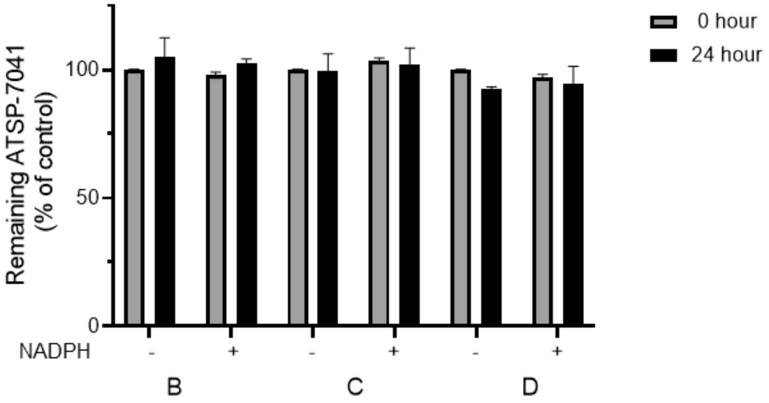

Figure S3. Vender differences in human liver S9 on metabolic stability of ATSP-7041.

ATSP-7041 (0.5 µM) was incubated in human liver S9 fractions (vender B, C, and D) (2 mg/mL) with or without NADPH at 37 °C for 24 h. Data are presented as % of the control (without NADPH, time 0 h) and mean ± S.D. (n = 3). Time 0 h (■) and 24 h (■).

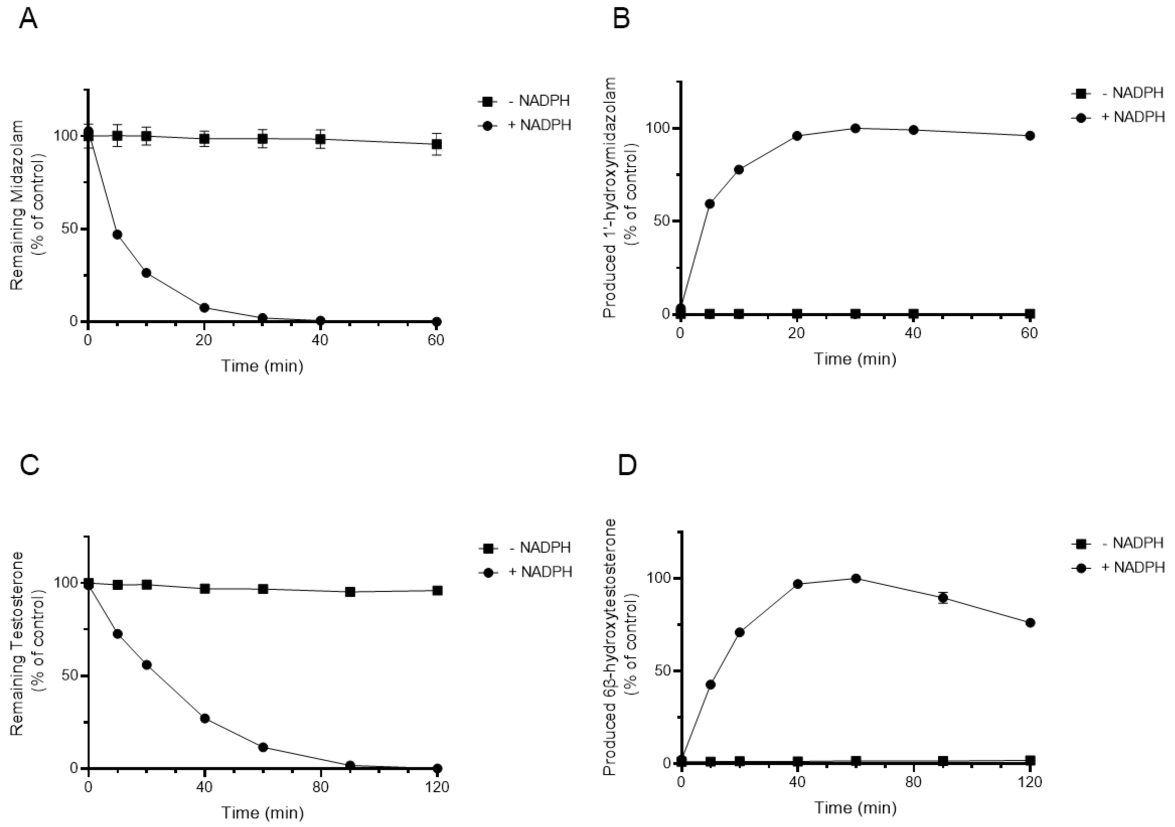

Figure S4. Time course in metabolism of midazolam and testosterone in human liver S9 fraction.
Midazolam (A) and 1'-hydroxylmidazolam (B) detected by incubation of midazolam (0.5 µM); testosterone (C) and 6β-hydroxytestosterone (D) detected by incubation of testosterone (0.5 µM) in human liver S9 fraction (2 mg/mL) with (-●-) or without (-■-) NADPH at 37 °C. Values are plotted as % of control (A: without NADPH, time 0 min; B: with NADPH, time 30 min; C: without NADPH, time 0 min; D: with NADPH, time 60 min), mean ± S.D. (n = 3).

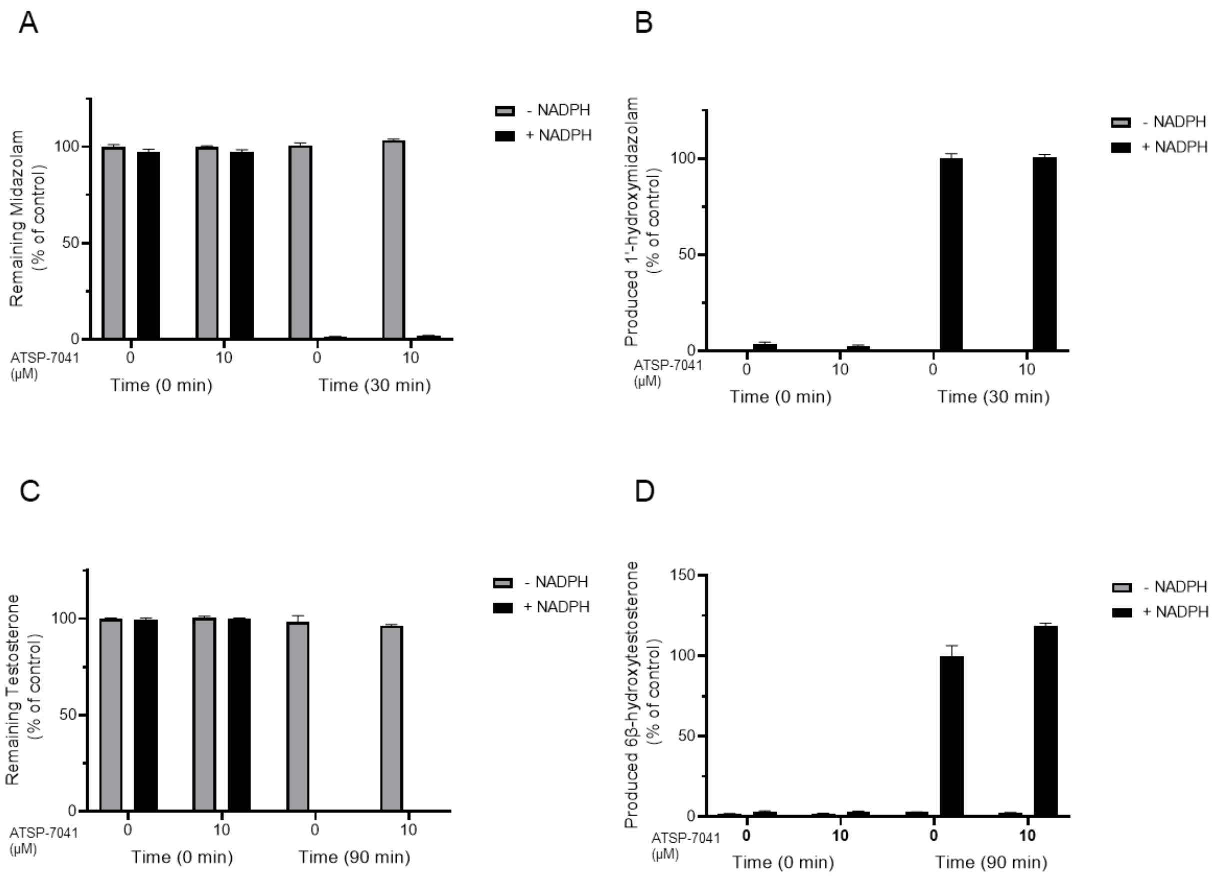

Figure S5. Effects of ATSP-7041 on the metabolism of CYP3A4 substrates in human liver S9 fraction.

Midazolam (A) and 1′-hydroxymidazolam (B) detected by incubation of midazolam (0.5 µM); and testosterone (C) and 6β-hydroxytestosterone (D) detected by incubation of testosterone (5 µM), with ATSP-7041 in human liver S9 fraction (2 mg/mL, vender A) with (-■-) or without (-■-) NADPH at 37 °C. Data are presented as % of control (A: without NADPH, time 0 min; B: with NADPH, time 30 min; C: without NADPH, time 0 min; D: with NADPH, time 90 min), mean ± S.D. (n = 3).

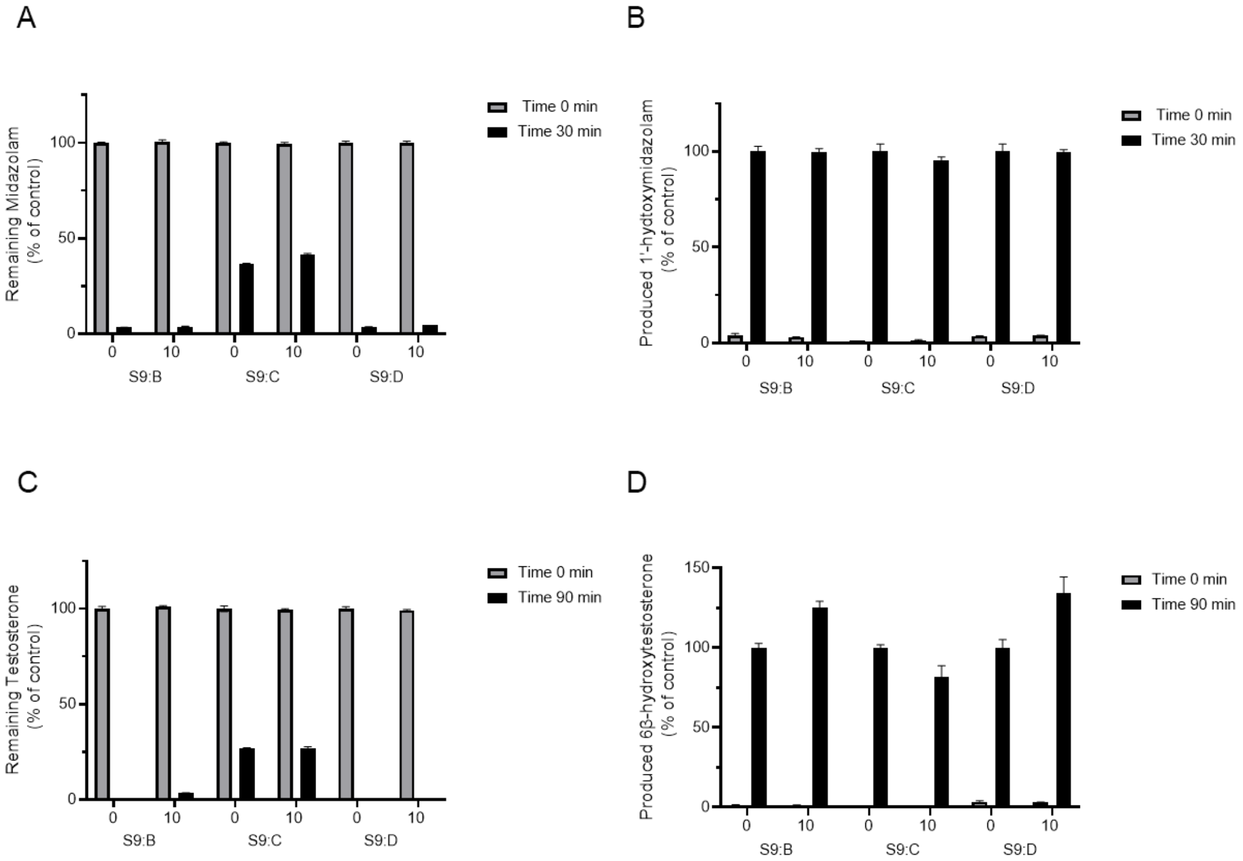

Figure S6. Vender differences in human liver S9 fraction in terms of effects of ATSP-7041 on metabolism of CYP3A4 substrates.

Midazolam (A) and 1’-hydroxymidazolam (B) detected by incubation of midazolam (0.5 µM) with ATSP-7041 in human liver S9 fraction (vender B, C, and D) (2 mg/mL) with NADPH at 37 °C for 30 min. Time 0 min (■) and 30 min (■). Values are plotted as % of control (A: time 0 min; B: time 30 min), mean ± S.D. (n = 3). Testosterone (C) and 6β-hydroxytestosterone (D) detected by incubation of testosterone (5 µM) with ATSP-7041 in human liver S9 fraction (2 mg/mL) with NADPH at 37 °C for 90 min. Time 0 min (■) and 90 min (■). Values are plotted as % of control (C: time 0 min; D: time 90 min), mean ± S.D. (n = 3).

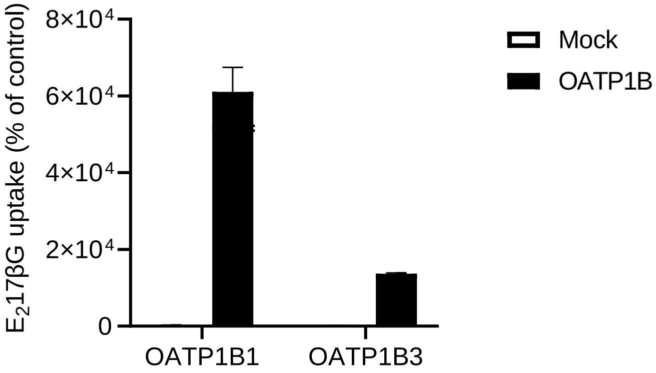

Figure S7. OATP1B-mediated uptake of E217βG.
Uptake of E217βG (1 μM) by OATP1B1 and OATP1B3-expressing HEK293 cells and mock cells. Data are presented as % of control (mock cells), the mean ± S.D. (n = 3).

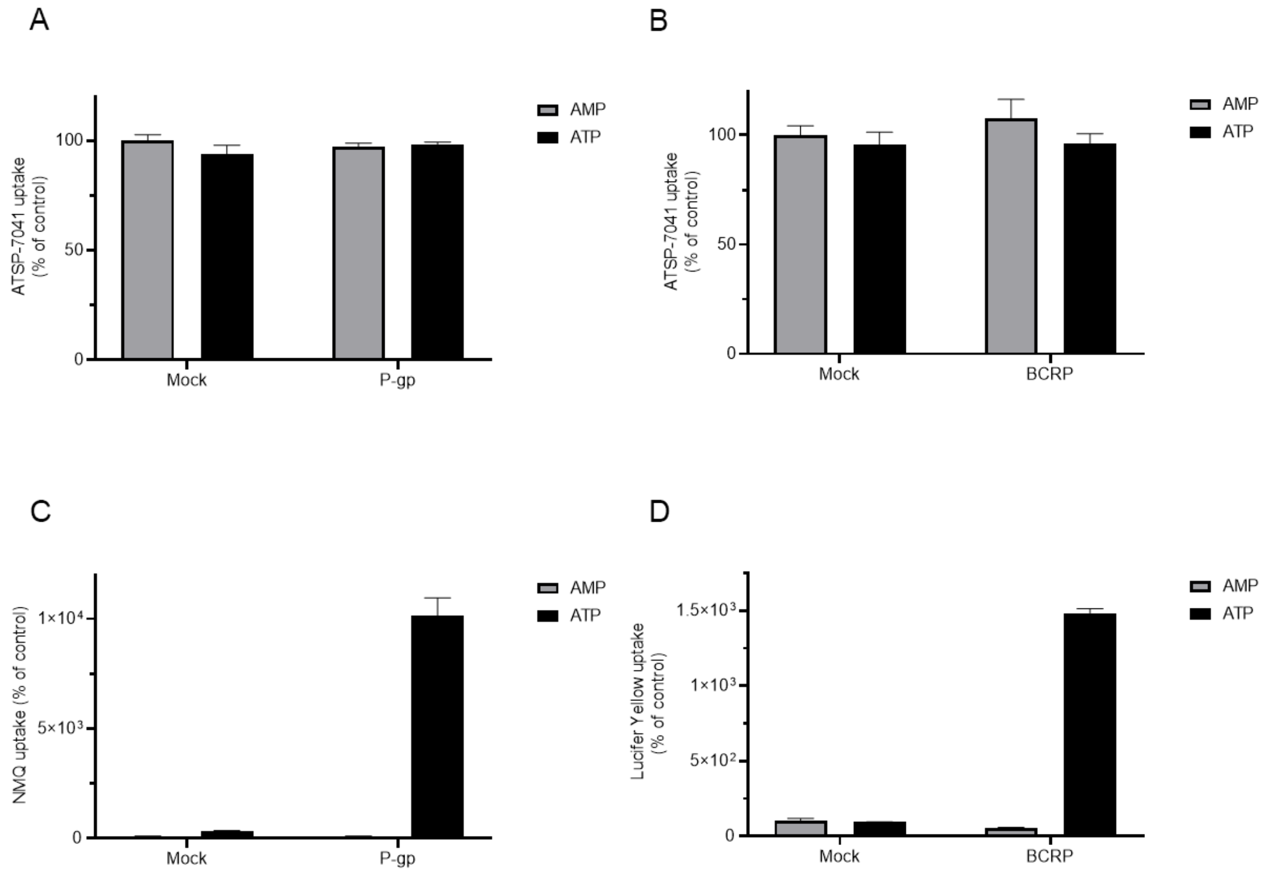

Figure S8. P-gp- and BCRP-mediated uptake of ATSP-7041.
Uptake of ATSP-7041 (0.1 μM) by reversed membrane vesicles from P-gp- (A) or BCRP-expressing (B) HEK293 cells and mock cells with AMP (■) or ATP (■). Uptake of NMQ (2 μM) (C) by reversed membrane vesicles from P-gp-expressing HEK293 cells and mock cells, and uptake of Lucifer yellow (50 μM) (D) by reversed membrane vesicles from BCRP-expressing HEK293 cells and mock cells with AMP (■) or ATP (■). Data are presented as % of control (mock cells, AMP), the mean ± S.D. (n = 3).
